# Effects of COVID-19 related physical inactivity on motor skills in children with intellectual disability

**DOI:** 10.1186/s12889-022-14887-y

**Published:** 2022-12-19

**Authors:** Parisa Sedaghati, Esmail Balayi, Somayeh Ahmadabadi

**Affiliations:** 1 Sports Injuries and Corrective Exercise, Faculty of Physical Education and Sports Sciences, University of Guilan, Rasht, Iran; 2 Sports injuries and corrective exercises- Adapted physical education, University of Guilan, Rasht, Iran; 3 Exercise Physiology, Department of Sports Sciences, Farhangian University, Tehran, Iran

**Keywords:** Coronavirus, Motor function, Postural control, Physical activity

## Abstract

**Background:**

Children with intellectual disabilities (ID) may show declines in motor skills during the Covid-19 restrictions. This study compared the effects of physical inactivity due to COVID-19 on the motor skills of active and inactive children with ID.

**Method:**

In this prospective cohort study, 30 boys with ID were divided into two groups based on study inclusion criteria (mean age 10.86 ± 1.81 active, 10.20 ± 1.42 inactive). The BESS test, the Y test, the Timed Up and Go (TUG) test, and the Bruininks-Oseretsky test-short form were used.

**Results:**

Results showed a significant difference between active and inactive groups in the total score of gross motor skills (*P* = 0.001), fine motor skills (*P* = 0.002), motor skills (*P* = 0.001), postural control (*P* = 0.01), and dynamic balance (*P* = 0.01).

**Conclusions:**

The results showed a significant difference between active and inactive children with ID in terms of gross and fine motor skills after a one-year movement restriction. Therefore, considering the tendency to be sedentary among these people and the subsequent complications caused by this inactivity, including obesity and chronic diseases, it is suggested that parents and educators design practical and numerous exercises and encourage them to be more active and participate in physical activity programs.

## Introduction

World Health Organization (WHO) declared a global pandemic on March 2020 because of the high number of deaths and infected cases caused by the coronavirus disease. Due to many problems resulting from the Covid-19 pandemic for the healthcare systems in different communities around the world [1] and to reduce the number of infections and deaths, countries ordered mass home-confinement directives including quarantine and physical separation. Staying at home and the resulting social isolation, are significant contributors that lead to widespread emotional distress resulting in issues such as sleep disorders and/or weakened immune systems [1, 2].

The prevalence of intellectual disabilities was 10.37/1000 population and low-and middle-income countries have been shown to have the highest rates [3]. In addition to a lower Intelligence Quotient (IQ) than the average of the community, children with ID are more physically inactive than other peers and are weaker in terms of physical fitness, strength, endurance, balance, and neuromuscular coordination [4], so that about 73 to 87% of these children have balance problems in daily activities [5]. Among the motor disabilities of these children, poor postural control is a major concern. On the one hand, maintaining the balance of these children, which is mostly dependent on their sense of sight, causes any sensory disturbance to result in falls, physical injuries, as well as restrictions on their movement and participation [6]. On the other hand, the postural control system plays an important role in individuals’ daily lives. Sensory information relies mostly on the content of postural work to obtain more accurate information about the position of different parts of the body and the body’s center of mass in space [7]. Maintaining muscle strength and endurance, and dynamic balance to achieve a better life and functional independence are also important factors in these individuals [8]. Previous studies reported low levels of physical fitness and high rates of obesity along with less participation in social activities, and cognitive decline in individuals with ID [9]. Moreover, children with ID have been shown to suffer from a range of chronic health conditions such as epilepsy, cerebral palsy, anxiety disorder, oppositional defiant disorder, Down syndrome, and autistic disorder [10].

In addition, given the above-mentioned studies, it can be said that despite their natural appearance, these children perform weaker in terms of gross or fine motor skills than their same-age peers [11]. These children usually have difficulty with gross or fine motor skills, and sometimes both of them, have slower and less accurate motor performance and are very different from their peers. Some children may have difficulty with the movement of their fingers and eye-hand coordination, and some may have poor balance [12]. These children have disorders in performing coordinated movements that make it difficult for them to perform specialized sports skills. Also, without any neurological diseases or special medical problems, they have coordination problems that affect their academic and social performance [13]. Therefore, balance, postural control, and motor skill components are potentially important in the lives of people with ID. The physical fitness of these individuals is defective compared to healthy individuals. Besides, they need to perform sports movements because the lack of physical fitness leads to obesity, limited motor function, decreased mobility and postural stability, and frequent falls [14].

Progressive changes at the community level have culminated in various inactive forms of physical activities and leisure, which have led to the formation of an inactive lifestyle. Meanwhile, some vulnerable groups, including people with ID and the elderly, have experienced the most harm and damage due to lifestyle changes. The occurrence of some international crises has always overshadowed the level of physical activity in people with ID. In such a condition, the COVID-19 outbreak as an worldwide problem has caused serious damage to the physical fitness, balance, motor skills, and exercise of individuals with disabilities and intellectual disorders across the world [15]. Meanwhile, home quarantine and school closures have affected children’s lifestyles due to less physical activity, more TV watching, and changes in bedtime. Such negative health effects are exacerbated when children are deprived of outdoor activities and interaction with their peers during a disease outbreak [16]. During home quarantine, closures and abnormal lifestyles have led many individuals in the community to stay home. Prolonged quarantines will inevitably cause psychological and socioeconomic reactions, physical inactivity, obesity, reduced components of physical fitness (balance, power, speed, etc.), and problems in gross and fine motor skills [17–19].

Children and youngsters have shown noticeable decreases in physical activity during the COVID-19 pandemic. The results of the research emphasize that the necessary conditions and sufficient support should be provided for physical activity to ensure good health and social functioning among children and adolescents during the pandemic recovery efforts [20]. In another study in the United Kingdom, George et al. reported the positive effect of COVID-19 restrictions on positive behavioral support for people with ID [21]. Nicolatis et al. (2021) investigated the effect of COVID-19-related restrictions on physical activity and the mental health of children and adults with physical and mental disabilities. The results of this study showed a 61% reduction in physical activity levels and more than 90% of negative effects on mental health. Many subjects mentioned lack of access to specialized, therapeutic, and necessary facilities and equipment as the reason for these negative effects. They also expressed concerns about the long-term effects of the restrictions on different levels of mental health and physical activity [22]. Sedaghati et al. (2021) investigated the effect of 1 year of physical inactivity due to COVID-19 on the motor function of the elderly living in care centers. According to the findings of the present study, elderly care centers and families with elderly people should pay close attention to the need for physical activity and exercise during this critical period for these individuals. Besides, in addition to planning to increase their levels of physical activity, they should be encouraged to participate in physical activities [23]. According to the instructions of the World Health Organization (WHO), the recommendation for healthy physical activity behavior for children and adolescents is at least 60 min of moderate-to-vigorous physical activity (MVPA) every day [24].

Since various dimensions of the consequences of COVID-19 in society are still unknown, and the factors exacerbating or weakening them are not yet fully understood, it seems necessary to conduct further research in this regard to better understand the reaction of different individuals in society, especially people with ID who are vulnerable to the consequences of COVID-19, to examine the state of motor skills, balance, and coordination in these individuals in interaction with the lifestyle in this era. Therefore, in this study, the effects of 1 year of physical inactivity due to COVID-19 on gross and fine motor skills, balance, and postural control of active and inactive children with ID were investigated.

## Materials and methods

This research is a prospective cohort study. Participants included boys with ID studying at Golestan Institution in the academic year (2020–2021). After obtaining permission from the Department of Education and making necessary arrangements, personal information and medical records of the subjects were collected using the files of children with ID. In this study, active children with ID were those who had regular physical activity for three sessions a week (36 sessions, one-hour physical activity in moderate intensity) under the supervision of a coach for 3 months before COVID-19 restrictions, and inactive individuals had no regular physical activity schedule before and after the restrictions.

**Fig. 1:**
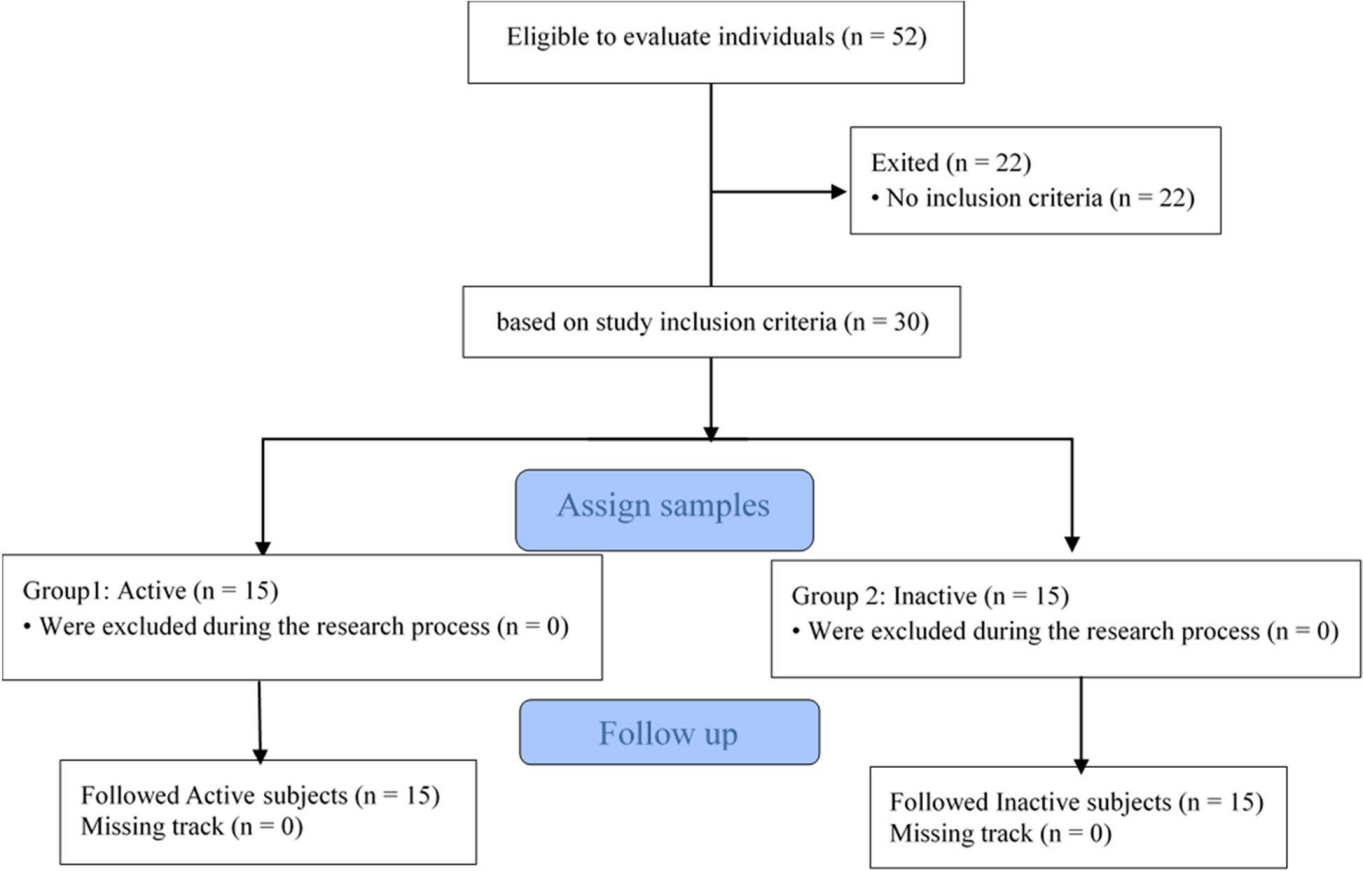
Flowchart study of protocol

The physical activity level was defined in-self-report according to the WHO physical activity guidelines during the COVID-19 pandemic that the movement restriction was determined by not having moderate-vigorous regular physical activity (i.e., walking and running) for 30 min to an hour even 1 day a week. Inclusion criteria included not participating in regular physical activity during the past year according to parents’ reports, taking neuroleptics or medicines affecting balance, having no history of lower limb injury and surgery during the past year, having no disease in the atrial system, and no cochlear implantation, no visual impairment, having a normal vision without glasses, willingness and ability to participate in the test, and parental informed consent to participate in the study. Exclusion criteria included ankle injuries, lower limb and spine surgeries during the past year, a history of neuromusculoskeletal diseases, severe hearing and vision problems, taking neuroleptics, and a history of lower limb injury and surgery. Then, written informed consent was obtained from the subjects’ parents for participation in the study (Fig. 1).

To determine the sample size, G*POWER statistical software was used with test power (0.80), effect size (0.50), and significance level (0.05)8, 15. The required number of samples was determined to be 30. Then, from among 50 educable boys with an age range of 6 to 13 years and an IQ between 50 and 75 based on the inclusion and exclusion criteria, 30 individuals were randomly selected through a purposeful and convenience sampling method and were randomly divided into two groups of active and inactive individuals.

Subjects were normalized in terms of age, height, weight, and Body Mass Index (BMI) in the desired groups. Subjects were evaluated in the pre-test regarding postural control, functional balance, dynamic balance, and motor skills using the BESS test, the Y test, the Timed Up and Go (TUG) test, and the Bruininks-Oseretsky test-short version in two stages, once in September 2020 and then a year later in 2021. The tests were taken by 2 researchers of this study in the indoor sports hall under the supervision of school authorities. All participants were evaluated during all research stages in terms of health protocols related to Covid-19.

### Postural control evaluation

The BESS test was used to measure static postural control with 75% validity and 50–82% reliability [25, 26]. The static balance test was performed in three positions: Standing position, including two legs next to each other, standing on one leg (the superior leg and the non-superior leg), and tandem standing position (standing with trailing leg, 1 foot forward, and one leg back). The superior leg was determined to measure balance tests by the tendency to shoot a soccer ball [27]. The hands were on the waist in all three positions, and the test was performed with the eyes closed. All three positions were performed on two hard (ground) and soft (foam) surfaces for 20 seconds for each position. The test time was recorded by a timer immediately after the subject’s eyes were closed. When performing the test in each position, six types of errors, if made, were counted and recorded for each subject. These errors included removing the hands from the waist, opening the eyes, stepping or falling, raising the heel or toe, flexing the torso forward or sideway more than 30 degrees, and deviating from the defined position, which was repeated for each position for 5 seconds and each test three times [28, 29].

### Dynamic balance measurement

To evaluate dynamic balance, the Y balance test with 91% validity and 84% reliability was used [30, 31]. In this test, three directions (anterior, posterior-external, and posterior-internal) were drawn at an angle of 135 degrees from each other. Since this test has a significant relationship with length, to perform it and normalize the real information of the foot, the upper anterior iliac spine to the inner ankle was measured in a supine position to lying down on the floor [30]. Each subject practiced the test six times to learn how to perform it. The subject stood on 1 foot in the center of the sitting area with the other foot in the direction chosen by the examiner, performed the maximum achievement action without error, and returned to the original position. To eliminate the learning effect, each subject practiced each direction six times, each time with a 15-second rest. After a five-minute rest, the subject began the test in the direction randomly chosen by the examiner, and the examiner measured the contact point of the person’s foot to the center of the toe in centimeters. The test was repeated three times for each subject, and the best record was divided by the leg length and then multiplied by 100 to obtain the achievement distance in terms of the leg length percentage. In the event of an error with the foot in the center, the test was repeated. The subject performed each direction three times, and the mean of three attempts was considered as the dynamic balance score [32].

### Functional balance evaluation

The TUG test was used for this purpose. A chair without a handle, a stopwatch, and a three-meter distance was required to perform this test. The three-meter path started from the legs of the chair. The subject, sitting in his usual shoes and clothes, sat on a chair and leaned on the chair back. At the command of the examiner, the subject got up and walked the marked three-meter distance. After arriving at the end, he turned around and sat down on his chair. In total, he walked six meters. The duration of the test was recorded as the individual’s score in seconds, and it is worth noting that the record of this test was the mean of performing it three times, and its validity and reliability were reported as 91 and 99%, respectively [33]. Individuals whose time in the test was less than 20 seconds were considered to have good independent movement [34].

### The Bruininks-Oseretsky test evaluation

The Bruininks-Oseretsky test of Motor Proficiency, 2nd ed. (BOT-2) is a set of tests specific for children aged 4.5–14.5 years that assesses gross and fine motor skills to identify their motor proficiency and motor disorder [35]. Based on the test instructions, preparation of testing conditions needs 10 minutes and Implementation of the full form takes 40–60 minutes. The BOT-2 examines 53 items in four motor area composites. Fine Manual Control (15 Items), which covers motor skills including control and coordination of the distal musculature of the hands and fingers; Manual Coordination (12 Items), coordination of the arms and hands, especially for object manipulation; Body Coordination (16 Items), coordination of the large musculature used in maintaining posture and balance; and Strength and Agility (10 Items), which consists of aspects of fitness and coordination involved in casual play, competitive sports, and other physical activity [36]. The raw score of each item is recorded in the unit measured (e.g. seconds, number of catches) and then converted into a numerical score [35]. In this study, the Bruininks-Oseretsky test was used to assess the gross and fine motor skills involving eight components including running speed and agility, balance, mutual coordination, strength, response speed, visual-motor control, and upper limb speed and agility in children with ID with developmental coordination disorders. These components measure four subscales of gross motor skills, three subscales of fine motor skills, and one subscale of both gross and fine motor skills [37]. This test has the necessary validity and reliability so that the validity coefficient of the Bruininks-Oseretsky test scores in the assessment of motor skills has been equal to 90%. The retest reliability coefficient of this collection has been reported to be 0.78 in the full form and 0.86 in its short form. The short-form version measures children’s motor skills in general, and the total score indicates children’s general skills, including gross and fine skills [38]. The short form of the test was used in this research.

The Shapiro-Wilk test was used to check the normality of data, and the Paired-Samples T-Test, Wilcoxon Signed Ranks Test, analysis of covariance (ANCOVA), and Mann–Whitney U was used to analyze the pre-test and post-test results at a significance level of *p* < 0.01in SPSS version 21 software.

## Results

Demographic characteristics of the subjects in two groups of active and inactive children with ID are reported in Table 1. The Paired-Samples T-Test (for parametric variables) was used to examine the difference between the pre-test and post-test in the two groups separately, which showed a significant difference in the total score of postural control, dynamic balance, functional balance, running speed and agility, balance static, strength, gross motor skills, motor visual control, the speed of agility and upper limbs, fine motor skills scores separately, as well as the total score of motor skills (*p* < 0.01) (Table 2).

**Table 1:** Demographic characteristics of the study samples

Measurement index	Group	Mean ± SD	T	P
**Age (y)**	**Inactive**	10.20 ± 1.42	1.22	0.27
	**active**	10.86 ± 1.81		
**Height (m)**	**Inactive**	1.39 ± 0.10	1.34	0.08
	**active**	1.44 ± 0.10		
**Weight (kg)**	**Inactive**	38.92 ± 9.75	2.12	0.19
	**active**	43.33 ± 10.80		
**BMI (kg/m2)**	**Inactive**	19.45 ± 2.65	0.27	0.78
	**active**	19.78 ± 3.84

**Table 2:** The difference between the mean of the variables in the before and after one-year limit physical activity

group	inactive group (15 n)				active group (15 n)			
	Pre-test	Post-test	T	pvalue	Pre-test	Post-test	T	pvalue
Variable								
The total score of postural control (number of errors)	6.35 ± 1.57	6.83 ± 1.30	−3.49	0.004^**^	3.45 ± 1.12	5.61 ± 1.1	−9.29	0.001^**^
Dynamic balance (cm)	46.21 ± 14.00	44.9 ± 14.44	9.06	0.001^**^	65.12 ± 14.6	58.75 ± 13.8	3.69	0.001^**^
Functional balance (s)	7.73 ± 1.17	7.01 ± 0.93	−3.6	0.003^**^	6.3 ± 0.71	8.16 ± 1.01	−4.93	0.001^**^
Running speed and agility	4.87 ± 2.53	4.27 ± 2.37	3.15	0.007^**^	6.39 ± 2.19	5.2 ± 2.27	11.31	0.001^**^
the balance static	1.93 ± 1.03	1.47 ± 0.83	3.5	0.004^**^	6.13 ± 1.24	4.73 ± 1.33	6.59	0.001^**^
the power	7.07 ± 2.49	6.4 ± 2.52	3.59	0.003^**^	10.7 ± 1.84	8.67 ± 2.32	6.81	0.001^**^
Gross motor skills score	15.13 ± 5.1	13.57 ± 5.19	4.94	0.001^**^	27.13 ± 3.98	21.26 ± 5.1	9.52	0.001^**^
Motor vision control	2.4 ± 1.45	1.67 ± 175	4.79	0.001^**^	5.27 ± 0.88	3.93 ± 1.03	10.58	0.001^**^
Speed of agility and upper limbs	4.8 ± 3.18	4.53 ± 2.95	2.57	0.04	10.47 ± 1.41	8.33 ± 2.32	6.56	0.001^**^
Fine motor skills score	8.18 ± 5.68	8.19 ± 5.68	4.12	0.002^**^	16.1 ± 1.92	7.39 ± 5.49	7.62	0.001^**^
The total score of motor skills	27.9 ± 10.9	23.92 ± 10.2	8.51	0.001^**^	50.08 ± 5.02	37.1 ± 6.54	14.99	0.001^**^

a Paired-Samples T Test - ** Significance at the level of P < 0.01

Wilcoxon test (non-parametric variable) was used to examine the differences in pre-test and post-test in the two groups separately, revealing a significant difference in the two-way coordination test and response speed between the two groups (p < 0.01) (Table 3).

**Table 3:** (mean difference) of different Two-way coordination and Response speed in subjects before and after one-year limit physical activity

group	Inactive group (15 n)				active group (15 n)				Comparison variables between groups	
Variable	Pre-test	Post-test	Z	pvalue	Pre-test	Post-test	Z	pvalue	Z#	pvalue
**Two-way coordination**	1.46 ± 0.74	1.07 ± 0.8	−2.53	0.01^**^	3.6 ± 0.63	2.67 ± 0.72	−3.54	0.001^**^	−4.06	0.001^**^
**Response speed**	0.07 ± 0.15	0.58 ± 0.11	−2.03	0.04	0.74 ± 0.55	0.39 ± 0.28	−2.95	0.001^**^	−4.21	0.001^**^

a Wilcoxon Signed Ranks Test- #Mann–Whitney U-** Significance at the level of P < 0.01

To compare the mean values between the two groups, considering the pre-test as a covariate, the covariance test was used, which showed a significant difference in the scores of postural control, dynamic balance, functional balance, running speed, and agility, balance static, strength, gross motor skills, motor visual control, the speed of agility and upper limbs, fine motor skills separately, as well as the total score of motor skills (*P* < 0.01) (Table 4).

**Table 4:** Comparison of variables between groups after one-year limited physical activity

Variable	F	df	*p*-value^#^	Eta Squared
The total score of postural control (number of errors)	6.83	1	0.01^**^	0.32
Dynamic balance (cm)	6.39	1	0.01^**^	0.19
Functional balance(s)	6.03	1	0.02	0.18
Running speed and agility	14.42	1	0.001^**^	0.35
the balance static	0.01	1	0.92	0.000
the power	8.96	1	0.006^**^	0.25
Gross motor skills score	12.86	1	0.001^**^	0.32
Motor vision control	0.09	1	0.77	0.003
Speed of agility and upper limbs	8.06	1	0.008^**^	0.23
Fine motor skills score	12.3	1	0.002^**^	31.8
The total score of motor skills	24.85	1	0.001^**^	0.49

# Analysis of covariance, a Pre-test (covariate agent), ** Significance at the level of P < 0.01

The non-parametric Mann–Whitney U test was used to examine the differences between the control and experimental groups given the abnormality of the two-way coordination score and response speed, which showed a significant difference in the two-way coordination test and response speed (P < 0.01).

## Discussion

This study aimed to investigate the effect of 1 year of physical inactivity due to COVID-19 on motor skills and postural control of active and inactive children with ID. The results of this study showed that the postural control, balance, and motor skills of active and inactive children with ID under the influence of a one-year COVID-19 decreased compared to before the epidemic. This decrease can be due to the imposed motor restrictions, home quarantine, and physical inactivity during this period. Besides, due to the prolonged motor restrictions in both active and inactive groups, we observed decreased motor function, postural control, and balance.

Since a few studies have examined the detrimental effects of motor restrictions due to the COVID-19 pandemic, the lack of comprehensive data on children with ID makes it more difficult to monitor the impact of COVID-19 on this population and identify risk factors. In this regard, Nicola Tiss et al.’s (2021) study examined the effects of COVID-19-related restrictions on physical activity and the mental health of children and adults with physical and mental disabilities. The results of this study showed a 61% reduction in the levels of physical activity and more than 90% of negative effects on mental health. Many subjects mentioned a lack of access to specialized, therapeutic, and necessary facilities and equipment as the reason for these negative effects. They also expressed concerns about the long-term effects of this restriction and disease on different levels of mental health and physical activity [22]. Sedaghati et al. (2021) reported that the period of motor restriction due to COVID-19 could decrease balance and motor function in the elderly and increase the likelihood of their falling [23]. In another study, Okechukwu et al. (2019) investigated the effects of quarantine and loneliness on the physical and mental health of the elderly during COVID-19. The results of this study showed that social and communication quarantine had a significant negative effect on the emotional, mental, and physical health of the elderly and led to reduced life expectancy. They also suggested that to prevent these negative effects on these individuals during COVID-19, the elderly should engage in activities such as sports, playing with peers, and recreation [39]. Most people with ID show a disturbed balance, a condition that causes significant delays in motor development and limits their functional levels. This balance restriction impairs movement in people with ID and reduces their physical initiative, both of which can lead to long periods of physical inactivity and a sedentary lifestyle in these individuals. Since COVID-19 poses challenges to maintaining an active lifestyle, it can lead to increased physical inactivity and ultimately, obesity and related diseases due to staying more at home [20].

Also, the decrease in motor skills and brain function was found to be significant in combination with a lack of physical fitness in manipulation, accuracy of movement, and motor activity [40]. Moreover, the deterioration in motor skills as measured by BOT-2 score test demonstrated a significant correlation with higher values of adiposity markers; BMI, WHR, oxidative stress parameters, and lower physical fitness scores in schoolchildren with mild and moderate ID compared with control group of standard IQ which consistently supported previously [9, 41]. Although general quarantine as a preventative measure against the spread of the disease reduces the spread of virus infection, it can reduce the level of physical activity as well. In other words, staying home, reducing social activities, and not doing physical activities such as walking, going to the park, and doing sports and recreation such as going to the pool, etc., are limitations that lead to worsened mood, feeling lonely, reduced interpersonal relationships, and consequently being at risk of chronic diseases and physical inactivity in these individuals [42]. In addition, it can impair the coordinated performance of movements or neuromuscular coordination so these disorders in coordination also cause a decline in their academic and social performance [13]. Therefore, safe independent mobility is important for participating in the community and daily activities of people with ID [43]. Because the decrease in motor development is related to the low level of physical activity, parents and caregivers of these children should encourage and support these students in more physical activity [44].

Therefore, the related authorities, in cooperation with families who have children with ID, by using sports consultants and experts, should provide individual consultations on the proposed exercise programs appropriate to the physical condition, functional abilities, and personal goals of people with ID in social restriction conditions. In some systems, these consultations can be performed using virtual applications to reduce the risks associated with face-to-face contact [45]. Receiving unique programs in person from a sports expert in schools will not always be practical for these individuals. Therefore, resources should be available to these individuals so that through which they can select the necessary exercises to maintain their physical health in this critical period and perform them continuously. The best tool for this purpose is online resources such as video training, which are relatively easy and cheap to publish. Therefore, organizations, departments, families, and health professionals should support the provision of these services to keep children with ID always active.

The limitations of this study include the relatively small sample size, the lack of control over the subjects’ mental and psychological states, the lack of access to the female group, the lack of control over the night activities, and the amount of sleep. It is suggested that future studies investigate the effects of COVID-19-related restrictions on the physical activity of other special individuals (Down syndrome, autism, hyperactive children, etc.) with a larger sample size. The present study did not receive any funding from government, private, or non-profit organizations.

### Perspective

The COVID-19 outbreak has led to problems in all aspects of human life. This disease also has caused serious damage to the health of people with ID and decreased their level of physical activity, physical fitness, general fitness, and free movement in them [27]. Thus, a lack of attention to sports culture, physical fitness, balance, and gross and fine motor skills of children with ID following the COVID-19 outbreak can have long-term physical and social effects in different communities. The lack of comprehensive research in this field has led to the lack of serious solutions designed and implemented by sports authorities today.

## Conclusion

The longitudinal study showed decreases in levels of postural control, balance, and motor skills under the influence of a one-year COVID-19 due to the imposed motor restrictions and home quarantine. Therefore, the related authorities in cooperation with families should provide children with necessary exercises such as online resources and video training to maintain their physical health in this critical period.

## Data Availability

The datasets generated and/or analyzed during the current study are available from the corresponding author on reasonable request.

## References

[1] Dergaa I, Ammar A, Souissi A, Fessi MS, Trabelsi K, Glenn JM, Ghram A, Taheri M, Irandoust K, Chtourou H (2022). COVID-19 lockdown: impairments of objective measurements of selected physical activity, cardiorespiratory and sleep parameters in trained fitness coaches. EXCLI J.

[2] Musa S, Elyamani R, Dergaa I (2022). COVID-19 and screen-based sedentary behaviour: systematic review of digital screen time and metabolic syndrome in adolescents. PLoS One.

[3] Maulik PK, Mascarenhas MN, Mathers CD, Dua T, Saxena S (2011). Prevalence of intellectual disability: a meta-analysis of population-based studies. Res Dev Disabil.

[4] Draheim CC, Williams DP, McCubbin JA (2003). Cardiovascular disease risk factor differences between special Olympians and non-special Olympians. Adapt Phys Act Q.

[5] Sabzi AH, Pak D, Taha. (2019). G The effect of twelve sessions of atrial stimulation exercises on balance function in children with developmental coordination disorder. J Rehabil Med.

[6] Fong SSM, Ng SMS, Chung MY, Ki W, Macfarlane DJ. Direction-specific impairment of stability limits and falls in children with developmental coordination disorder. In: International Society of Physical and Rehabilitation Medicine (ISPRM) 2016; 2016. 10.1016/j.gaitpost.2015.10.026 26669953

[7] Horak FMJ. Handbook of physiology: a critical, comprehensive presentation of physiological knowledge and concepts. New York Oxford Am. Physiol Soc. 1996:92–255.

[8] Kajbaf M, Mansour M, Ejei J, Parirokh D (2000). Survey and diagnosis of mental retardation based Piaget tests and Lambert scale. Psychology.

[9] Alghadir AH, Gabr SA (2020). Physical activity impact on motor development and oxidative stress biomarkers in school children with intellectual disability. Rev Assoc Med Bras (1992).

[10] Oeseburg B, Dijkstra GJ, Groothoff JW, Reijneveld SA, Jansen DEC (2011). Prevalence of chronic health conditions in children with intellectual disability: a systematic literature review. Intellect Dev Disabil.

[11] Emerson E, Einfeld S, Stancliffe RJ (2010). The mental health of young children with intellectual disabilities or borderline intellectual functioning. Soc Psychiatry Psychiatr Epidemiol.

[12] Zwicker JG, Missiuna C, Harris SR, Boyd LA (2011). Brain activation associated with motor skill practice in children with developmental coordination disorder: an fMRI study. Int J Dev Neurosci.

[13] Carmeli E, Bar-Yossef T, Ariav C, Levy R, Liebermann DG (2008). Perceptual-motor coordination in persons with mild intellectual disability. Disabil Rehabil.

[14] Eslamdost M, Sheikh M, Mohammadi M, Ahmadi G (2017). The effect of core stability training on static and dynamic balance in children with developmental coordination disorder. Community Health Journal.

[15] Hammami A, Harrabi B, Mohr M, Krustrup P (2019). Physical activity and coronavirus disease (COVID-19): specific recommendations for home-based physical training. Manag. Sport Leis.

[16] Liu JJ, Bao Y, Huang X, Shi J, Lu L (2020). Mental health considerations for children quarantined because of COVID-19. Lancet Child Adolesc Health.

[17] Musa S, Dergaa I (2022). A narrative review on prevention and early intervention of challenging behaviors in children with a special emphasis on COVID-19 times. Psychol Res Behav Manag.

[18] Trabelsi K, Ammar A, Masmoudi L, Boukhris O, Chtourou H, Bouaziz B, Brach M, Bentlage E, How D, Ahmed M (2021). Globally altered sleep patterns and physical activity levels by confinement in 5056 individuals: ECLB COVID-19 international online survey. Biol Sport.

[19] Trabelsi K, Ammar A, Masmoudi L, Boukhris O, Chtourou H, Bouaziz B, Brach M, Bentlage E, How D, Ahmed M (2021). Sleep quality and physical activity as predictors of mental wellbeing variance in older adults during COVID-19 lockdown: ECLB COVID-19 international online survey. Int J Environ Res Public Health.

[20] Neville RD, Lakes KD, Hopkins WG, Tarantino G, Draper CE, Beck R, et al. Global changes in child and adolescent physical activity during the COVID-19 pandemic: a systematic review and Meta-analysis. JAMA Pediatr. 2022. 10.1001/jamapediatrics.2022.2313 PMC9274449 35816330

[21] Murray GC, McKenzie K, Martin R, Murray A (2021). The impact of COVID-19 restrictions in the United Kingdom on the positive behavioural support of people with an intellectual disability. Br J Learn Disabil.

[22] Theis N, Campbell N, De Leeuw J, Owen M, Schenke KC. The effects of COVID-19 restrictions on physical activity and mental health of children and young adults with physical and/or intellectual disabilities. Disabil Health J. 2021:101064. 10.1016/j.dhjo.2021.101064 PMC7825978 33549499

[23] Sedaghati P, Tabatabai Asl SM, Rahimi Moghaddam SR. The effect of one year of inactivity caused by Covid-19 on the motor function of the elderly living in care centers. J Rehabil Med. 2021.

[24] World Health Organization T (2010). Global Recommendations on Physical Activity for Health.

[25] Bell DR, Guskiewicz KM, Clark MA, Padua DA (2011). Systematic review of the balance error scoring system. Sports Health.

[26] Azadbakht H, Yousefi M, Azadi M. The effect of 12 weeks of core stability training on the balance of mental retardation children aged 9 to 11 years. In: First National Conference on New Achievements in Physical Education and Exercise Chabahar University; 2015.

[27] Daneshmandi H, Barati AH, Ahmadi R (2013). The effect of core stabilization training program on the balance of mentally retarded educable students. Arch. Rehabil..

[28] Blomqvist S (2013). Postural balance, physical activity and capacity among young people with intellectual disability.

[29] Kubilay NS, Yildirim Y, Kara B, Harutoglu Akdur H (2011). Effect of balance training and posture exercises on functional level in mental retardation. Fiz. Rehabil..

[30] Clark M, Fater D, Reuteman P (2000). Core (trunk) stabilization and its importance for closed kinetic chain rehabilitation. Orthop. Phys. Ther. Clin..

[31] Babakhani F (2020). Effectiveness of central muscle exercises with Physioball ball on balance and lordosis curvature change in educable mentally retarded female students. J. Health Res..

[32] McGill SM, Childs A, Liebenson C (1999). Endurance times for low back stabilization exercises: clinical targets for testing and training from a normal database. Arch Phys Med Rehabil.

[33] Kim Y, Kim HA, Kim J-H, Kim Y, Lim Y (2010). Dietary intake based on physical activity level in Korean elementary school students. Nutr Res Pract.

[34] Shumway-Cook A, Brauer S, Woollacott M (2000). Predicting the probability for falls in community-dwelling older adults using the timed up & go test. Phys Ther.

[35] Deitz JC, Kartin D, Kopp K (2007). Review of the Bruininks-Oseretsky test of motor proficiency, (BOT-2). Phys Occup Ther Pediatr.

[36] Venetsanou F, Kambas A, Aggeloussis N, Serbezis V, Taxildaris K (2007). Use of the Bruininks–Oseretsky test of motor proficiency for identifying children with motor impairment. Dev Med Child Neurol.

[37] Hale L, Bray A, Littmann A (2007). Assessing the balance capabilities of people with profound intellectual disabilities who have experienced a fall. J Intellect Disabil Res.

[38] Bruininks RH, Bruininks BD (2005). Bruininks-Oseretsky test of motor proficiency.

[39] Okechukwu CE (2021). The impact of loneliness on physical and mental health among older adults in the era of coronavirus disease 2019 pandemic. Apollo Medicine.

[40] Golubović ŠMJ, Golubović B, Glumbić N (2012). Effects of exercise on physical fitness in children with intellectual disability. Res Dev Disabil.

[41] Lotan M, Isakov E, Kessel S, Merrick J (2004). Physical fitness and functional ability of children with intellectual disability: effects of a short-term daily treadmill intervention. TheScientificWorldJOURNAL.

[42] Moro T, Paoli A. When COVID-19 affects muscle: effects of quarantine in older adults. Eur J Transl Myol. 2020;30(2). 10.4081/ejtm.2019.9069 PMC7385699 32782767

[43] Enkelaar L, Smulders E, van Schrojenstein Lantman-de Valk H, Geurts AC, Weerdesteyn V (2012). A review of balance and gait capacities in relation to falls in persons with intellectual disability. Res Dev Disabil.

[44] Wouters MEH, Hilgenkamp TIM (2019). Physical activity levels of children and adolescents with moderate-to-severe intellectual disability. J Appl Res Intellect Disabil.

[45] Vetri L, Elia M, Vitello GA, Greco D, Gagliano C, Costanzo MC, et al. Impact of daytime routine modifications on people with severe intellectual disability amid COVID-19 pandemic. Perspect Psychiatr Care. 2021. 10.1111/ppc.12696 PMC8242444 34032288

